# TOXIC CHEMICAL EMISSIONS AND DEMENTIA RISK: RESULTS FROM THE CARDIOVASCULAR HEALTH COGNITION STUDY

**DOI:** 10.1093/geroni/igad104.1188

**Published:** 2023-12-21

**Authors:** Breanna Crane, Kyle Moored, Patrick Donahue, Anne Corrigan, Frank Curriero, Andrea Rosso, Gina Lovasi, Michelle Carlson

**Affiliations:** Johns Hopkins Bloomberg School of Public Health, Baltimore, Maryland, United States; Johns Hopkins Bloomberg School of Public Health, Baltimore, Maryland, United States; Johns Hopkins University, Baltimore, Maryland, United States; Johns Hopkins Bloomberg School of Public Health, Baltimore, Maryland, United States; Johns Hopkins Bloomberg School of Public Health, Baltimore, Maryland, United States; University of Pittsburgh, Pittsburgh, Pennsylvania, United States; Drexel University Dornsife School of Public Health, Philadelphia, Pennsylvania, United States; Johns Hopkins Bloomberg School of Public Health, Baltimore, Maryland, United States

## Abstract

Air pollution is a modifiable risk factor for dementia. Yet, studies on specific forms of air pollution (e.g., toxic chemical emissions from industrial facilities) and dementia risk are underexplored. We examined associations between toxicity-weighted concentrations of industrial pollution and dementia outcomes among a large, multi-site cohort of older adults. Participants (n=2,770) were ≥ 65 years old (M=75.3, SD=5.1 years) from the Cardiovascular Health Cognition Study. Toxicity-weighted concentrations were estimated using the Risk Screening Environmental Indicator (RSEI) model which incorporates total reported chemical emissions with toxicity, fate, and transport models. Estimates were aggregated to participants’ baseline census tract, averaged across 1988-1992, and log10-transformed. Dementia status was clinically adjudicated in 1998-1999 (M=5.2 person-periods) by subtype (all-cause, Alzheimer’s, vascular, mixed). We assessed whether RSEI-estimated toxicity-weighted concentrations were associated with: 1) odds of prevalent dementia and 2) incident dementia risk by subtype. After adjusting for individual and neighborhood-level covariates, a 1-unit increase in log10-toxicity was associated with a 34% increase in the odds of prevalent dementia (OR=1.34, 95% CI: 1.01, 1.78). In discrete-time survival models, each 1-unit increase in log10-toxicity was associated with a 65% increased hazard of vascular dementia (HR=1.65, 95% CI: 1.04, 2.61) but was not significantly associated with all-cause, Alzheimer’s disease, or mixed dementia. Living in regions with higher toxic air emissions was associated with greater odds of prevalent dementia and a heightened risk of incident vascular dementia. Results suggest that the RSEI model may be a novel screening tool for examining associations between toxic chemical releases and dementia risk.

